# Continuous Auditory Feedback Promotes Fine Motor Skill Learning in Mice

**DOI:** 10.1523/ENEURO.0008-25.2025

**Published:** 2025-03-05

**Authors:** Dongsheng Xiao, Matilde Balbi

**Affiliations:** Queensland Brain Institute, The University of Queensland, Brisbane, 4072 Queensland, Australia

**Keywords:** closed-loop system, continuous auditory feedback, machine learning, motor skill learning, real-time

## Abstract

Motor skill learning enables organisms to interact effectively with their environment, relying on neural mechanisms that integrate sensory feedback with motor output. While sensory feedback, such as auditory cues linked to motor actions, enhances motor performance in humans, its mechanism of action is poorly understood. Developing a reliable animal model of augmented motor skill learning is crucial to begin dissecting the biological systems that underpin this enhancement. We hypothesized that continuous auditory feedback during a motor task would promote complex motor skill acquisition in mice. We developed a closed-loop system using DeepLabCut for real-time markerless tracking of mouse forepaw movements with high processing speed and low latency. By encoding forepaw movements into auditory tones of different frequencies, mice received continuous auditory feedback during a reaching task requiring vertical displacement of the left forepaw to a target. Adult mice were trained over 4 d with either auditory feedback or no feedback. Mice receiving auditory feedback exhibited significantly enhanced motor skill learning compared with controls. Clustering analysis of reaching trajectories showed that auditory feedback mice established consistent reaching trajectories by Day 2 of motor training. These findings demonstrate that real-time, movement-coded auditory feedback effectively promotes motor skill learning in mice. This closed-loop system, leveraging advanced machine learning and real-time tracking, offers new avenues for exploring motor control mechanisms and developing therapeutic strategies for motor disorders through augmented sensory feedback.

## Significance Statement

Enhancing motor skill learning can greatly improve therapeutic options for patients suffering from motor disorders. Our study demonstrates that continuous, movement-coded auditory feedback markedly accelerates complex motor skill acquisition in mice. By providing real-time auditory cues linked to specific forepaw movements through a closed-loop system—without the need for invasive markers—this approach offers a novel method for investigating the neural mechanisms of motor learning in neuroscience. It also opens new avenues for developing therapeutic strategies for motor function rehabilitation.

## Introduction

Motor skill learning is a fundamental aspect of behavior that enables organisms to interact effectively with their environment. This process involves the acquisition and refinement of movements through practice and experience, relying on complex neural mechanisms that integrate sensory feedback with motor output to produce coordinated actions (Shmuelof and Krakauer, 2011). Understanding the factors that enhance motor skill learning is crucial not only for basic neuroscience but also for developing therapeutic strategies for motor disorders.

Augmented sensory feedback, particularly auditory and visual cues linked to motor actions, has been shown to play a significant role in motor learning and rehabilitation. In humans, studies have demonstrated that enhanced sensory feedback can improve motor performance in tasks requiring fine motor control and coordination (Sigrist et al., 2013; Effenberg et al., 2016). However, the mechanism underlying augmented motor skill learning remains poorly understood.

Rodent models are an invaluable tool for investigating the neural circuits underlying motor learning due to the availability of advanced genetic and neurophysiological tools (Costa, 2011). However, classical rodent motor tasks, such as the rotarod test, the staircase test (Montoya et al., 1991), or single-pellet skilled reaching paradigms, generally rely on post hoc scoring and do not offer real-time, continuous feedback based on specific motor actions. As a result, these approaches limit our ability to systematically investigate how augmented feedback influences motor skill acquisition in real-time.

Advances in computer vision and machine learning have led to the development of tools capable of high precision, markerless tracking of animal movements. DeepLabCut, a deep learning framework for pose estimation, has enabled researchers to track specific body parts of freely behaving animals with remarkable accuracy (Mathis et al., 2018). This technology opens new avenues for delivering motor feedback in real-time, allowing us to develop reliable animal models of augmented motor skill learning to investigate how real-time, continuous movement-coded sensory feedback influences the learning of complex motor skills in rodents.

We hypothesize that continuous auditory feedback, coded in real-time based on the learner’s own forepaw movements, can enhance the acquisition of complex motor skills in mice. By providing immediate and specific sensory cues linked to motor actions, we aim to engage sensorimotor integration processes more effectively, leading to accelerated learning and improved motor performance.

In this study, we developed a high-speed (>70 Hz), low-latency (∼30 ms delay) closed-loop system built on the DeepLabCut framework to provide real-time auditory feedback to mice during a reaching task. By encoding forepaw movements into auditory tones of different frequencies, we provided continuous feedback that corresponds directly to the mice's motor actions.

Our objective was to determine whether movement-coded auditory feedback enhances motor skill learning in mice. By addressing this objective, we aim to contribute to the understanding of sensorimotor learning mechanisms and to develop methodologies that could inform therapeutic strategies for motor function rehabilitation.

## Materials and Methods

### Animals

Adult male C57BL/6 mice (8–10 weeks old, weighing 20–25 g) were used in this study. Mice were housed individually in a temperature-controlled environment with a 12 h light/dark cycle (lights on at 7:00 A.M.) and had *ad libitum* access to food and water unless otherwise specified.

All experimental procedures were approved by the Animal Ethics Committee of the University of Queensland.

### Experimental setup

#### Real-time tracking system

We developed a real-time tracking system based on the DeepLabCut framework (Mathis et al., 2018) for markerless pose estimation, which builds on recent advances in low-latency closed-loop feedback using markerless posture tracking (Kane et al., 2020) and selective markerless tracking of rodent forepaws (Forys et al., 2020), ensuring robust and efficient real-time feedback. The system was optimized for high-speed processing (>70 Hz) and low-latency feedback (∼30 ms delay). Modifications to DeepLabCut include multithreaded processing and GPU acceleration using an NVIDIA Titan Xp graphics card to ensure rapid frame acquisition and analysis.

High-speed video of the mice was captured using a USB3 Vision camera (Model STC-MCCM401U3V, Omron Sentech) at a resolution of 640 × 480 pixels, up to 360 fps. The camera was positioned to provide a clear front view of the mouse's forepaws during the reaching task. Video acquisition was handled using custom Python scripts interfacing with the camera's SDK, ensuring synchronization with the tracking and feedback systems.

#### Auditory feedback system

The auditory feedback system was designed to provide continuous, real-time auditory cues corresponding to the vertical displacement of the mouse's left forepaw. Specifically, we implemented tone frequencies in the 2–20 kHz range, as the speaker system used in our setup is rated for reliable sound production across these frequencies. Although mice can detect ultrasonic frequencies up to ∼100 kHz (Heffner and Heffner, 2007), generating robust ultrasonic tones above 20 kHz would have required hardware beyond our system's specifications.

Notably, mice also exhibit a significant sensitivity peak for audible sounds in the 10–20 kHz range (Heffner and Heffner, 2007). Therefore, operating between 2 and 20 kHz still overlaps substantially with frequencies to which mice are most sensitive. Forepaw movements were linearly mapped onto this frequency range using the PyAudio library in Python and delivered through a speaker placed ∼20 cm from the mouse.

To maintain a rapid feedback loop, we integrated audio generation and playback directly into the main real-time processing pipeline. Specifically, once the pose estimation algorithm detects a movement, a low-latency audio callback is immediately triggered to generate and play the corresponding auditory tone. This approach bypasses additional buffering or thread-switching overhead by handling both pose estimation (on the GPU) and audio output in a tightly coupled, multithreaded environment. As a result, the total end-to-end latency—from movement detection to auditory cue—is kept within 30 ms. The control group did not receive any auditory feedback during the task.

#### Behavioral task and training protocol

At the start of each training session, mice were habituated to the head-fixed apparatus in a custom-designed acrylic tube that secured their heads while allowing free movement of the forelimbs. Mice were positioned with a waterspout near their mouth and trained to lift their left forepaw from an initial resting position to a target zone (∼15 mm above the starting point). This target zone was defined in the real-time pose estimation software based on forepaw pixel coordinates. A “successful reach” was counted only when the left forepaw traversed from the designated start area into the target zone. Upon successful completion of each reach, a water reward (5 µl) was delivered via a precision liquid dispenser positioned near the mouse's mouth. For consistency, the forepaw had to return to the initial resting position before the next trial could begin.

To provide a clear contextual cue without acting as a primary reinforcer, a low-intensity green LED was placed near the animal's field of view but angled away so as not to directly illuminate its eyes. When illuminated, the LED signified that a training block was active; when turned off, the training session was paused or ended. This continuous signal was used instead of flashing lights or other transient cues to avoid startle responses or unintended behavioral modulation. Importantly, both the auditory feedback group and the control group were exposed to the same LED conditions, ensuring that any minor visual influence from the LED was identical across experimental conditions.

Mice underwent daily training sessions lasting 30 min for 4 consecutive days. Prior to the training sessions, mice were water-restricted to motivate participation in the task. Water restriction was carefully managed to maintain at least 85% of the animals' baseline body weight, and mice received supplemental water as needed to meet this criterion. Each training session included multiple trials, with mice allowed to initiate reaching attempts at their own pace. For the auditory feedback group (*n* = 7), every detected upward displacement of the left forepaw triggered an auditory tone (2–20 kHz) scaled linearly to the paw's vertical position. This continuous, movement-linked tone provided augmented sensory feedback during the reach. The control group (*n* = 7) performed the same reaching task but did not receive any auditory feedback. All other aspects of the task—housing conditions, experimenter handling, and water restriction protocols—remained the same between groups, ensuring that any difference in motor skill acquisition could be primarily attributed to the presence or absence of continuous, movement-coded auditory feedback.

The green LED served as a state indicator for the training paradigm. When illuminated, it signified that the training session was active, whereas its absence indicated baseline recording. This constant visual cue helps the animal recognize the training context without providing an external reward or disruptive stimulus. To limit direct illumination into the mouse's eyes, the LED was placed at ∼10 cm from the animal's head. The LED's luminous intensity was set to <1 cd, based on in-lab measurements at this distance, providing a low-intensity cue. This arrangement reduces the influence of the LED on the animal's behavior while remaining perceptible as a contextual signal.

### Data collection

#### Video and audio recording

High-speed video recordings were captured at a user-selectable frame rate (up to 360 fps), but higher frame rates demand more computing power, which can increase overall processing delay in the closed-loop system. Consequently, we typically limited frame rates to a level (∼70 fps) that balanced tracking accuracy with minimal latency for real-time feedback (Forys et al., 2020). In addition to the primary camera used for tracking, a secondary camera recorded the overall behavior video and audio to capture the timing and frequency of the auditory tones. This allowed for synchronization of movement data with the auditory feedback for analysis.

#### Movement tracking

The tracking system identified and tracked eight digits of the mice's left and right forepaws. The DeepLabCut model was trained using a dataset of labeled images capturing various paw positions and movements. A general model of mouse paw movement was trained based on ResNet-50by labeling 1,000 frames selected using k-means clustering (Nath et al., 2019) to sample a variety of movement dynamics from one video of each of the 11 mice recorded.

### Data analysis

#### Processing of movement data

The vertical displacement of the left forepaw was extracted from the tracking data. Reaching trajectories were compiled for each session, and movements were aligned temporally to the initiation of each reach.

#### Principal component analysis and t-SNE

To analyze the complexity and consistency of reaching movements over time, we applied principal component analysis (PCA) to the reaching trajectories. PCA reduced the dimensionality of the data, allowing us to identify the main principal components contributing to the variance in the trajectories. Additionally, t-distributed stochastic neighbor embedding (t-SNE) was used to visualize high-dimensional movement data in a two-dimensional space. This nonlinear dimensionality reduction technique allowed us to cluster similar reaching movements and observing changes in movement patterns over the training period.

#### Clustering

A Gaussian mixture model (GMM) was employed to cluster the reaching trajectories based on their features extracted from PCA analyses. The number of clusters was determined using the Bayesian information criterion, selecting the model that best fit the data without overfitting.

#### Statistical analysis

Statistical analyses were performed using Python's SciPy and StatsModels libraries. Behavioral metrics were compared between groups using two-way repeated-measures ANOVA with factors for group (auditory feedback vs control) and day of training. Significance was set at *p* < 0.05. Effect sizes were calculated using Cohen's *d* to assess the magnitude of differences between groups.

#### Software and hardware specifications

Hardware:

Camera: Omron Sentech STC-MCCM401U3V USB3 Vision Camera

Graphics Card: NVIDIA Titan Xp GPU

Computer: Custom-built PC with Intel Core i7 processor, 32 GB RAM

Speakers: High-frequency response speakers (20 Hz–20 kHz range)

Microphone: Condenser microphone for audio recording

Software:

Operating System: Windows 10 Pro 64-bit

DeepLabCut Version: 2.1

Python Version: 3.7

Libraries: NumPy, SciPy, OpenCV, PyAudio, scikit-learn, Matplotlib

Custom Scripts: Developed for real-time tracking, auditory feedback generation, and data analysis

#### Ethical considerations

All procedures involving animals were conducted in compliance with the ethical standards and guidelines for the care and use of laboratory animals. Efforts were made to minimize the number of animals used and their discomfort throughout the experiments. Mice were monitored daily for health and well-being, and water restriction protocols were implemented carefully to avoid undue stress. All mice tolerated the head fixation and water restriction protocols without any signs of distress or adverse effects.

## Results

**Figure 1.**
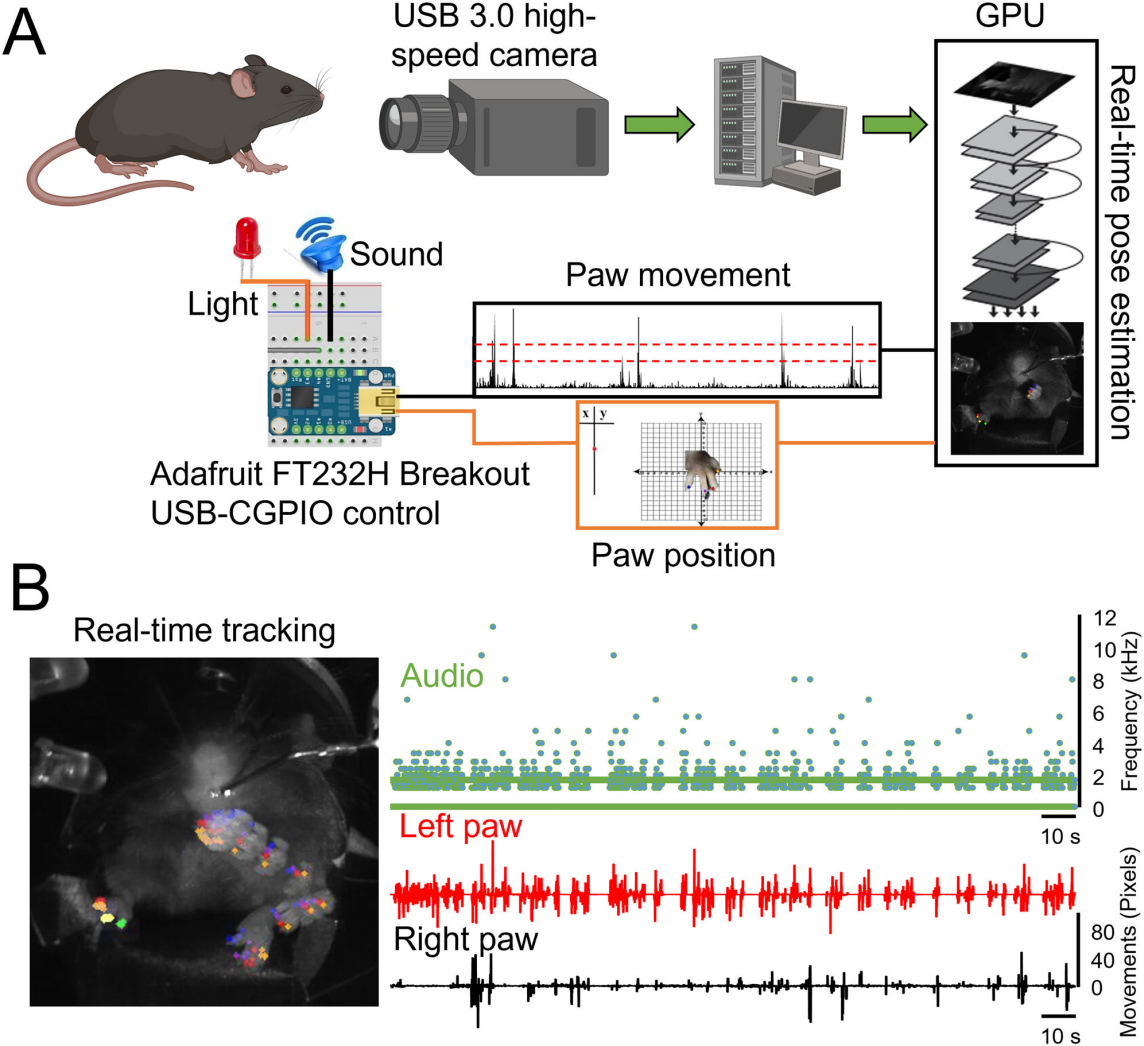
Experimental setup and real-time tracking system for providing continuous auditory feedback during a reaching task in mice. ***A***, Schematic diagram of the closed-loop system integrating real-time pose estimation and auditory feedback. A high-speed camera captures the mouse's forepaw movements, which are processed by the deep neural network to track paw digits. The vertical displacement of the left forepaw is encoded into auditory tones delivered through a speaker, providing immediate feedback to the mouse. ***B***, Left panel, An example image showing real-time tracking of the forepaw digits. Right panel, Time series plots of the auditory tone frequency (green), left paw vertical displacement (red), and right paw vertical displacement (black). The auditory tone frequency corresponds closely with the left paw movement, illustrating the system's real-time feedback capability.

Our customized real-time tracking system (Fig. 1*A*) successfully monitored mouse forepaw movements with high accuracy and low latency. The system operated at an average processing speed exceeding 70 Hz, with a mean delay of ∼30 ms between movement detection and auditory feedback delivery. Figure 1*B* (left panel) illustrates an example of real-time tracking of the left forepaw during the reaching task. The right panel of Figure 1*B* shows the corresponding real-time auditory tone (green trace) alongside the left (red trace) and right (black trace) paw movements. In this setup, forepaw movements were encoded into auditory tones whose frequency (2–20 kHz) was mapped linearly to the vertical displacement of the left forepaw, thus providing continuous, movement-coded feedback.

Throughout the experiments, a low-intensity green LED was illuminated to indicate active training blocks for both the auditory feedback group and the control group. Because it served only as a contextual cue and was presented identically in both groups, the LED was unlikely to contribute differentially to motor skill acquisition. This aligns with previous findings in wide-field imaging paradigms that employ continuous LED illumination without influencing behavioral performance (Ma et al., 2016; Wekselblatt et al., 2016).

### Enhanced motor skill learning with continuous auditory feedback

**Figure 2.**
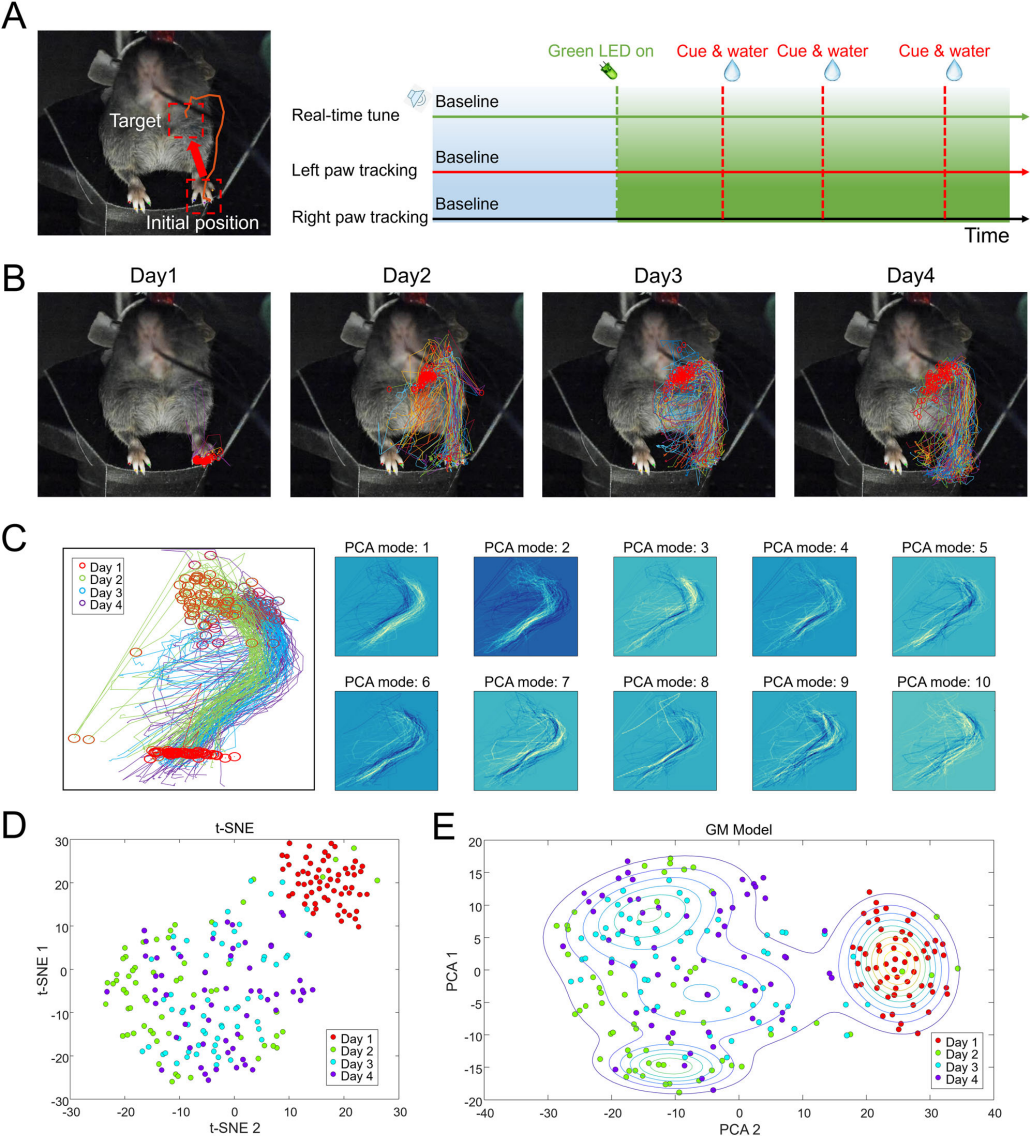
Clustering analysis of reaching trajectories during motor training. ***A***, Left panel, Diagram of the reaching task setup, where the mouse must move its left forepaw from the initial position to the target region (red square) to perform a successful reach. Right panel, Illustration of the training schedule over 4 d. ***B***, Reaching trajectories of the left forepaw for a representative mouse in the auditory feedback group over the four training days. Each plot overlays multiple reaching movements. ***C***, Principal component analysis (PCA) of reaching trajectories. ***D***, t-distributed stochastic neighbor embedding (t-SNE) plots of reaching trajectories. ***E***, Clustering of reaching trajectories using Gaussian mixture model (GMM).

Throughout the training sessions, mice in both groups engaged in the reaching task. We analyzed the reaching trajectories of mice over the 4 d training period to assess motor skill acquisition and refinement. Figure 2*A* (left panel) depicts the setup of the reaching task, with the target region indicated by the red square. With continued auditory feedback, mice learned to reach to the target region by Day 2 (Fig. 2*B*). Clustering analysis of the reaching trajectories (Fig. 2*C*–*E*) revealed that the trajectories from Days 2, 3, and 4 largely formed a single cluster, indicating that mice established relatively consistent reaching motions by Day 2 and maintained those consistent patterns through Days 3 and 4.

We performed PCA on the reaching trajectories to identify the principal components contributing to movement variance. Mice receiving auditory feedback developed stereotyped and efficient reaching movements over time (Fig. 2*C*). t-SNE analysis (Fig. 2*D*) provided a nonlinear dimensionality reduction to visualize the clustering of reaching trajectories. Using Gaussian mixture model (GMM) clustering, we further analyzed the reaching trajectories (Fig. 2*E*). We observed that the trajectories from Days 2, 3, and 4 consistently formed a cluster distinct from Day 1, indicating that the mice had already established stable reaching patterns by Day 2 and maintained them through subsequent training days. This finding supports the notion that task learning occurred as early as Day 2.

### Auditory feedback significantly increases reaching motions

**Figure 3.**
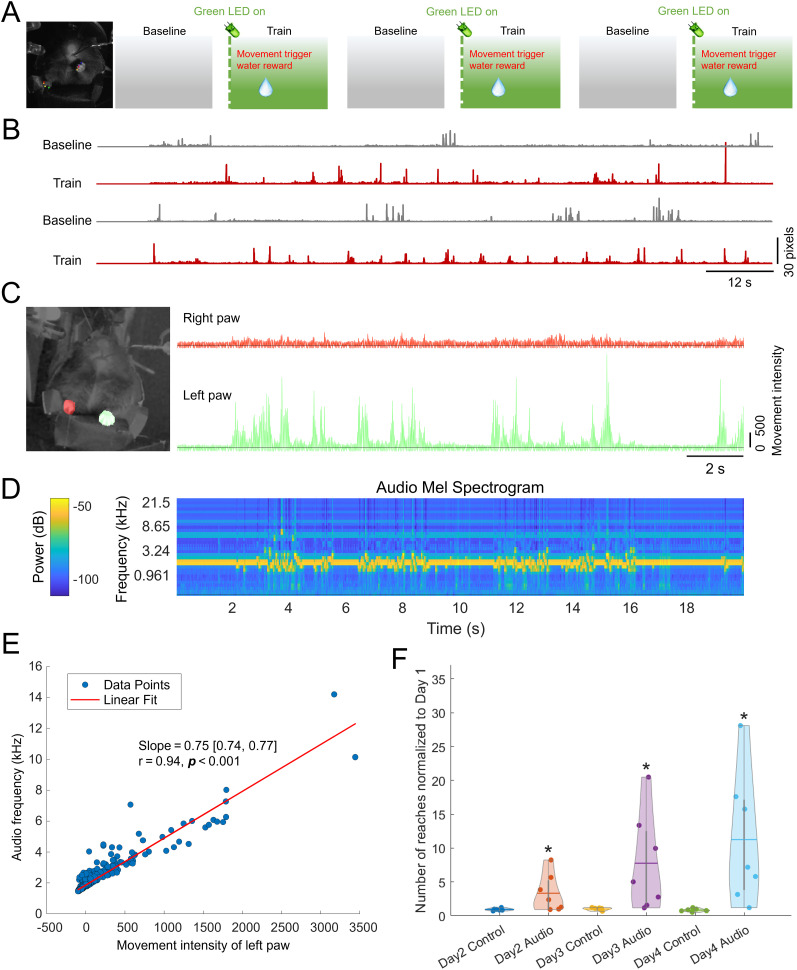
Continuous movement-coded auditory feedback promotes motor skill learning. ***A***, Schematic of the experimental setup comparing the auditory feedback group and the control group. Mice in the auditory feedback group received real-time auditory tones corresponding to their left forepaw movements during the reaching task. Both groups performed the same reaching task under the same low-intensity green LED illumination, which served solely as a contextual cue indicating active training. ***B***, Example plots of left forepaw vertical displacement during baseline (gray) and training (red) for a mouse in the auditory feedback group. ***C***, Left, Image of a mouse during the motor training session (video and audio are recorded by a second camera). Right panel, Plot of forepaw movements over time during a training session. ***D***, Mel spectrogram of the real-time audio recorded by the second camera. ***E***, Scatter plot showing the correlation between audio frequency and left paw movement intensity (Pearson's *r* = 0.94; slope = 0.75 [0.74, 0.77], two-tailed *t* test *p* < 0.001). The red line represents the best-fit linear regression. ***F***, Comparison of motor learning between the auditory feedback group (*n* = 7) and the control group (*n* = 7) over 4 d of training. Violin plots represent the ratio of left paw reaches to right paw reaches, normalized to Day 1. The auditory feedback group demonstrated significantly higher reach ratios on Days 2, 3, and 4 (asterisks denoting *p* < 0.05). Unpaired two-sample *t* tests for each day revealed: Day 2 Control versus Audio (*p* = 0.0428), Day 3 Control versus Audio (*p* = 0.0289), and Day 4 Control versus Audio (*p* = 0.0144). A two-way repeated-measures ANOVA (day × group) revealed significant main effects of group (*p* < 0.05), as well as a day × group interaction (*p* < 0.01), indicating that continuous auditory feedback alters the progression of motor skill learning over time.

The Mel spectrogram of the real-time audio recorded during the sessions shows frequency changes that align with the movements of the paw, indicating that mice received immediate and continuous feedback on their movements (Fig. 3). To compare overall motor learning between the auditory feedback and control groups over 4 training days, we analyzed the normalized number of reaches, calculated as the ratio of left paw reaches to right paw reaches and normalized to reaches in Day 1. By Day 4, the auditory feedback group exhibited a significantly higher reach ratio than the control group (*p* < 0.05; Fig. 3*F*), suggesting that movement-coded auditory feedback effectively promoted motor skill learning relative to the control condition.

Our results demonstrate that continuous real-time auditory feedback of motion trajectories significantly enhances the acquisition and refinement of motor skills in mice. The analyses using PCA, t-SNE, and GMM clustering provide evidence that auditory feedback facilitates the development of more refined and consistent motor patterns. These findings support the hypothesis that continuous sensory feedback linked to specific motor actions effectively promotes motor skill learning.

## Discussion

In this study, we investigated whether continuous, movement-coded auditory feedback could enhance the learning of complex motor skills in mice. Our findings demonstrate that mice receiving real-time auditory feedback linked to their forepaw movements exhibited significantly accelerated motor skill acquisition compared with control mice without feedback. This aligns with previous research in humans, where augmented sensory feedback has been shown to improve motor performance and learning efficiency (Sigrist et al., 2013; Effenberg et al., 2016). The auditory system's high temporal resolution may allow for rapid processing of feedback, enabling mice to make immediate adjustments to their movements. By providing a direct and continuous link between the motor action and sensory consequence, the auditory feedback likely enhanced the sensorimotor integration processes essential for skill acquisition.

The clustering of reaching patterns, performed by using PCA, t-SNE, and GMM analyses, indicates that auditory feedback helped mice consolidate their movements into more efficient and stereotyped patterns. This refinement of motor strategies is a hallmark of motor skill learning, where practice leads to the optimization of neural circuits controlling the movement (Dayan and Cohen, 2011). While our study did not directly examine the neural substrates involved, it is plausible that the enhanced motor learning observed with auditory feedback is mediated by changes in the cortico-basal ganglia-thalamocortical loops, which are critical for motor skill acquisition and habit formation (Graybiel, 2008; Costa, 2011). The auditory feedback may facilitate synaptic plasticity within these circuits by providing an additional sensory input that reinforces correct movements and aids in error correction. Moreover, this approach could be extended or complemented by other sensory modalities (e.g., visual or haptic feedback), further augmenting motor learning and offering new avenues for rehabilitation and research into sensorimotor integration.

Importantly, our use of a low-intensity green LED as a continuous state indicator allowed us to standardize the training context without conferring differential reinforcement. Because the same LED conditions were applied uniformly to both the auditory feedback and control groups, any potential influence of this visual cue on behavioral performance was minimized. The LED functioned to signal whether a training block was active or paused, thereby reducing the chance that mice would confuse trial availability. Unlike the continuous auditory feedback, however, the LED was not intended to serve as a reward or motivational stimulus—rather, it was purely a contextual marker. In contrast, the continuous movement-linked auditory cue may have offered a consistent reward-like signal that motivated mice to refine their forepaw movements more quickly. Previous studies have shown that augmented feedback, especially when sonified, can reinforce correct actions and heighten engagement in sensorimotor tasks (Huang et al., 2011; Sigrist et al., 2013; Effenberg et al., 2016).

Our findings demonstrate that continuous, movement-coded auditory feedback in the 2–20 kHz range enhances motor skill learning in mice. Although their auditory sensitivity extends to the ultrasonic range (∼100 kHz), mice retain a notable sensitivity peak within 10–20 kHz (Heffner and Heffner, 2007). Consequently, employing tones between 2 and 20 kHz enabled us to provide salient sensory feedback without the need for specialized ultrasonic hardware. Several rodent studies have similarly relied on audible frequencies in behavioral and associative learning paradigms (Fanselow, 1990; Phillips and LeDoux, 1992; LeDoux, 2000; Maren, 2001; Suga and Ma, 2003), supporting our study.

Moreover, the integration of auditory feedback with motor commands could engage the auditory cortex and its connections with motor regions. Studies have shown that multisensory integration can enhance neuronal responsiveness and improve behavioral outcomes (Kayser and Logothetis, 2007). Future studies employing electrophysiological recordings or imaging techniques could elucidate the specific neural adaptations associated with the observed behavioral enhancements.

Our findings have potential implications for developing therapeutic interventions for motor disorders where the natural feedback modalities—such as proprioception, vision, or tactile sensation—are impaired or partially lost. In such cases, providing an additional or alternative sensory channel can help compensate for deficient intrinsic feedback mechanisms. By encoding movement information into a modality that remains intact, it may be possible to facilitate error correction, enhance motor coordination, and promote neuroplasticity in pathways responsible for motor control. This concept could be extended to clinical scenarios such as stroke rehabilitation, Parkinson's disease, or spinal cord injuries, where augmenting or substituting damaged sensory pathways may improve patients’ ability to regain or refine motor skills.

The closed-loop system we developed confers several advantages for studying motor control and learning. First, real-time pose estimation using DeepLabCut is directly integrated into the feedback pipeline, allowing specific body part movements to be captured without markers or wearable sensors, thus preserving the animals’ natural movement patterns. Second, the low-latency architecture of our system ensures that auditory or other sensory cues are delivered quickly in response to detected movements—an essential factor for reinforcing the link between action and consequence. Finally, our modular design can be adapted to various forms of sensory feedback (e.g., visual or tactile) and different motor tasks, making it a versatile platform for a broad range of neuroscience and behavioral research paradigms.

Furthermore, our head-fixed preparation stabilizes the animal's viewpoint and body alignment, reducing motion artifacts and improving the precision of real-time tracking. Moving forward, combining our behavioral paradigm with neurophysiological measurements (e.g., electrophysiology or imaging) in a head-fixed context could further reveal the underlying neural changes that mediate motor skill learning and validate the proposed mechanisms. In the future, extending the system to freely moving animals could offer opportunities to study more naturalistic behaviors and complex motor tasks, albeit with additional technical challenges in tracking and stimulus delivery. Finally, exploring the effects of feedback in animal models of motor impairments would help evaluate the therapeutic potential of this approach in disease contexts such as stroke or Parkinson's disease. In conclusion, our study demonstrates that continuous auditory feedback of motion trajectories significantly enhances the learning of motor skills in mice. These findings contribute to the understanding of sensorimotor integration in motor learning and highlight the potential of augmented sensory feedback in developing therapeutic strategies for motor disorders. The closed-loop system we developed, leveraging advanced machine learning algorithms and real-time movement tracking, offers a powerful tool for neuroscience research. This animal model of augmented sensory feedback opens new avenues for exploring the neural mechanisms underlying motor control and learning and designing interventions that can enhance motor rehabilitation through targeted sensory feedback.

## Synthesis

Reviewing Editor: Christine Portfors, Washington State University Decisions are customarily a result of the Reviewing Editor and the peer reviewers coming together and discussing their recommendations until a consensus is reached. When revisions are invited, a fact-based synthesis statement explaining their decision and outlining what is needed to prepare a revision will be listed below. The following reviewer(s) agreed to reveal their identity: Jinxing Wei.

The reviewers agree that this is an interesting study requiring a few clarifications.

This study developed a closed-loop system using DeepLabCut for real-time tracking of mouse forepaw movements. By encoding forepaw actions into auditory tones, it provided continuous feedback during motor tasks. Results showed that auditory feedback significantly enhanced motor skill learning, offering insights into motor control mechanisms and potential therapeutic strategies for motor disorders.

Here are some suggestions to strengthen their claims.

1）The methods describing the behavioral training and testing processes are too simplistic. Moreover, the role of the green LED in the behavioral training and testing is not clarified in the results section or figure legends. Additionally, I am puzzled as to why the authors used a green LED. Compared to red light, the mouse visual system is more sensitive to green light. Could the LED light stimulus alone affect behavioral performance?

2）The authors selected a frequency range of 20 Hz-20 kHz, likely based on the frequency range perceptible to humans. However, the frequency sensitivity range in mice is significantly different from that of humans. The authors need to provide an explanation for using this specific frequency range.

3）In Fig. 3E, the authors should display the fitting parameters in the figure to help readers better understand the data.

4）In Fig. 3F, the authors only compared the normalized values of the control and auditory feedback groups on Day 4 relative to Day 1. To better understand the changes throughout the training process, the authors should include the normalized values for Day 2 and Day 3 relative to Day 1. Additionally, since statistical analyses were conducted and yielded significant results, the authors should indicate these statistical significances in the figure to enhance reader comprehension.
